# Association between bullying victimization and mental health problems among Chinese left-behind children: a cross-sectional study from the adolescence mental health promotion cohort

**DOI:** 10.3389/fpsyt.2024.1440821

**Published:** 2024-11-07

**Authors:** Yuan Feng, Simai Zhang, Xiao Liao, Yuge Jia, Yu Yang, Wei Zhang

**Affiliations:** ^1^ Mental Health Center, West China Hospital, Sichuan University, Chengdu, China; ^2^ West China Biomedical Big Data Center, West China Hospital, Sichuan University, Chengdu, China; ^3^ Med-X Center for Informatics, Sichuan University, Chengdu, China; ^4^ School of Management and the Key Laboratory of Process Optimization and Intelligent Decision-Making, Ministry of Education, Hefei University of Technology, Hefei, China

**Keywords:** depression, anxiety, self-injurious behavior, bullying, left-behind children (LBC)

## Abstract

**Background:**

Left-behind children (LBC) refer to those who have been separated from at least one parent for six months or more due to parental migration for work. This phenomenon poses a significant threat to the mental health of over 61 million LBC in China. This study aims to compare the prevalence of mental health symptoms between LBC and non-left-behind children (non-LBC) and to explore the predictive effect of bullying victimization on adolescent mental health problems.

**Methods:**

In 2019, we conducted a cross-sectional analysis involving 28,036 children and adolescents in Mianyang City, Sichuan Province, China, with ages ranging from 8 to 19 years. Mental health symptoms were assessed using the 7-item Generalized Anxiety Disorder (GAD-7) scale and the 9-item Patient Health Questionnaire (PHQ-9). The Delaware Bullying Victimization Scale-Student (DBVS-S) was employed to gather data on experiences of bullying victimization. Information on self-injury was collected by inquiring whether participants had engaged in self-injurious behavior and the reasons for such behavior. Multivariable logistic regression was utilized to analyze the risk and protective factors associated with mental health symptoms, with a particular focus on different types of bullying victimization.

**Results:**

Compared to non-left-behind children (non-LBC), left-behind children (LBC) exhibited a higher prevalence of mental health issues: anxiety symptoms (24.0% vs. 18.0%, p<0.001), depressive symptoms (27.9% vs. 19.4%, p<0.001), and self-injurious behavior (17.7% vs. 12.2%, p<0.001). Among LBC, physical bullying was identified as the most significant predictor of anxiety symptoms (OR = 1.62). Additionally, LBC who experienced verbal bullying had a higher risk of depressive symptoms (OR = 2.23) and self-injurious behaviors (OR = 1.54). Enhanced family functioning, positive teacher-student relationships, and strong peer relationships were found to offer protective effects against mental health problems.

**Conclusion:**

Our results suggested that LBC experienced a higher incidence of mental health symptoms, particularly among those who had been victims of bullying. This underscores the urgent need for supportive strategies focused on the school environment and interpersonal relationships to mitigate negative mental health outcomes for LBC.

## 1 Introduction

The urbanization and modernization since China’s Reform and Opening-up have led to a surge in migrant workers, resulting in many individuals moving from their hometowns to seek employment in cities (1). Consequently, a significant number of children and adolescents remain in their rural hometowns due to the high cost of living, education, and healthcare in urban areas. A nationwide survey in China (2014) reported that approximately 61 million children and adolescents were left behind, representing 22.0% of the total child population in China (2). Left-behind children (LBC) typically remain in their original residence for at least six months while one or both parents migrate for work (3, 4). Intuitively, the lack of care and supervision may heighten LBC’s risk for poor nutrition, accidents, and injuries (5). From a developmental psychology perspective, parental absence can impact children’s social development, emotional support, self-regulation, and the development of self-concept (6). Early-life adversity, such as parental absence, may lead to feelings of insecurity and sustained tension, affecting the development of the HPA axis (7, 8). This has a negative impact on the development of attachment relationships (9) and result in decreased emotional support and increased feelings of loneliness (10). Previous studies have also indicated that self-rated health status and parent-child relationships can vertically predict adolescent personality development (11, 12). Thus, parental absence can influence the development of self-identity and the formation of a healthy personality (13, 14). Furthermore, early victimization increases LBC’s susceptibility to mental illness (15, 16), making them more prone to externalized behavioral problems, internalized disorders, and inferiority complexes (17), such as hallucinations, delusions, and emotional problems (18–20). Compared to non-LBC, the incidence of mental health problems among LBC is over 10.0% higher (21). Given this high prevalence, it is crucial that researchers pay increased attention to the current state and risk factors affecting the mental health of LBC.

Bullying is a widespread issue affecting children and adolescents globally (22, 23). Bullying victimization manifests in two primary forms: traditional and cyberbullying. Traditional bullying victimization is characterized by harm inflicted through physical, verbal, or relational aggressive behavior from peers (24). In contrast, cyberbullying involves the deliberate use of electronic media to inflict harm (25). Globally, approximately 10.0% to 30.0% of adolescents experience bullying (26). A cross-sectional study conducted in China reported that the highest self-reported rate of traditional bullying victimization was 66.0%, while the peak rate of cyberbullying victimization was 57.0% (27). In recent years, the reported rates of bullying victimization among children and adolescents in China have ranged from 8.0% to 15.1% (28, 29).

As a traumatic experience, bullying victimization during primary and secondary school years can adversely affect mental health into early adulthood (30, 31). The extent and variety of bullying experienced are directly correlated with increased mental health harm (22). Bullied adolescents reported a range of emotional and behavioral problems, including anxiety (32), depressive symptoms (33), and avoidance behavior (34). A growing body of literature indicates that adolescents who experience bullying are at a heightened risk for developing anxiety and depressive symptoms (35). Consequences of bullying can also include low self-esteem, academic difficulties (36), increased substance abuse (37) and even suicidal ideation or behavior (38–40). Studies have shown that LBC experience higher rates of bullying and greater victimization scores compared to non-LBC (41). The unsafe attachment patterns often associated with being left behind can impair individual abilities and social development, making these children more vulnerable to bullying (42–44). Additionally, factors such as the main caregivers and the duration of parental separation are linked to the extent of bullying victimization (45).

Given the significant long-term impact of bullying on mental health, it is crucial to identify the key risk and protective factors influencing adolescents’ psychological well-being. This focus is vital for disease prevention and health promotion, particularly during adolescence. There is still limited understanding of how different types of bullying victimization relate to mental health outcomes and the protective factors that may mitigate these effects. Using cross-sectional data from students aged 8-19 in China, we aimed to explore the association between various types of bullying victimization and mental health problems, with a particular emphasis on the experiences of being left behind.

## 2 Methods

### 2.1 Participants

Sichuan, located in western China, is the fourth most populous province, with a total population exceeding 82 million. The Adolescence Mental Health Promotion Cohort is a prospective cohort including 29,768 children and adolescents recruited from 29 local schools through stratified randomization in Mianyang City, Sichuan Province, China, in 2019. The distribution of students across primary, middle, and high schools was relatively balanced. The overall participation rate was 74.0%, representing approximately 30.0% of students in Santai County. All participants were surveyed through a WeChat applet called Psyclub.

In our research, participants were asked whether they had been separated from their parents for at least six months during childhood (yes or no). Those who answered “yes” were categorized into the LBC group.

**Figure 1:**
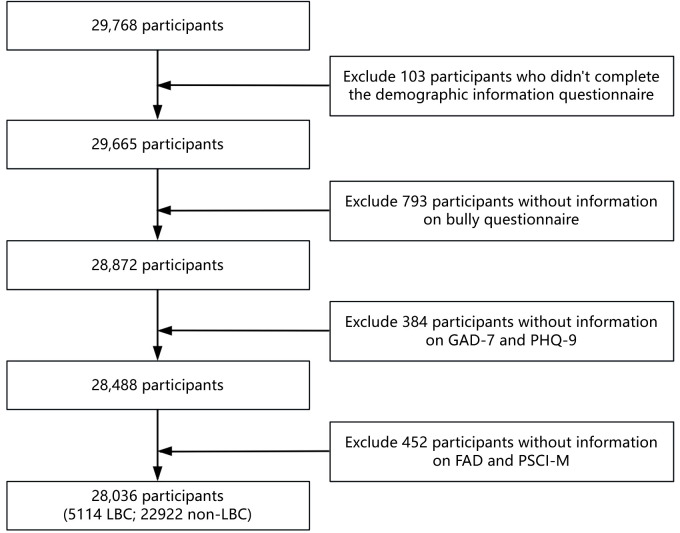
Sample inclusion flow chart.

Of the 29,768 participants, 103 were excluded due to incomplete demographic information, including 50 LBC who did not provide details about their primary caregiver during their stay. 793 participants were excluded for failing to complete the bullying exploration questionnaire. An additional 384 were excluded due to missing assessments for anxiety and depression symptoms. Another 452 were excluded from this study as they did not complete the questionnaires on family functioning and perceived school atmosphere. Ultimately, 5114 LBC and 22922 non-LBC were included in the study (**Figure 1**). Participants were aged 8 to 19 years, with a mean age of 13.5. All participants and their guardians reviewed the evaluation content, purpose, and electronic version of the informed consent form on Psyclub. They confirmed their agreement to participate in the study and signed the informed consent. The study protocol was approved by the Ethics Committee of West China Hospital, Sichuan University [2019-77].

### 2.2 Collection of self-reporting problem information

#### 2.2.1 Primary caregiver

We collected information on the primary caregivers of participants throughout their development (referred to as “caregivers” in the tables), as well as the primary caregivers of left-behind children (LBC) during their absence (referred to as “LBC caregivers” in the tables). Participants were asked via a questionnaire to identify who primarily cared for them during their growth, with options including both parents, father only, mother only, grandparents, and other close relatives. For participants who had not lived with their parents for more than six months, we additionally inquired, “Who primarily takes care of you when your father and/or mother are away?” The response options included father, mother, grandparents, siblings, other close relatives, and others.

#### 2.2.2 Self-injurious behavior

In the basic information questionnaire, participants were asked about their engagement in self-injurious behavior through self-reported questions. Specifically, we inquired, “Have you ever intentionally harmed yourself (e.g., through cutting, burning, or any other means)?” If participants reported self-injurious behavior, we further asked whether the intent behind such behavior was suicidal.

### 2.3 Bullying (DBVS-S)

The Delaware Bullying Victimization Scale-Student (DBVS-S) was employed to assess campus bullying victimization. The scale comprises 17 items: four items each for verbal bullying, physical bullying, social/relational bullying, and cyberbullying, plus a 13th item, “I was bullied at this school,” which is not included in the dimension scores but reflects the participant’s perception of being bullied (46). A six-point Likert scale was used to measure the severity of bullying, ranging from “never” (1 point) to “every day” (6 points). The Cronbach’s α for the DBVS-S was 0.906 (47). A score greater than 8 in any dimension was defined as “often,” and any occurrence of bullying was classified as “often” if at least one dimension met this criterion. The Cronbach’s α in our sample was 0.959.

### 2.4 Anxiety (GAD-7)

The 7-item Generalize Anxiety Disorder (GAD-7) scale was utilized to assess anxiety symptoms. The GAD-7 employs a Likert scale for self-evaluation, ranging from 0 (none at all) to 3 (almost every day). The total score ranges from 0 to 21 points, with higher scores indicating more severe anxiety symptoms. A score of 5 or above suggests the presence of anxiety symptoms, a score of 10 or above indicates moderate anxiety symptoms, and a score of 15 to 21 indicates severe anxiety symptoms (48). The Cronbach’s α of Chinese version of the GAD-7 scale was 0.915, and was 0.893 in our sample (49). In this study, a total score of 10 or higher was considered indicative of significant anxiety symptoms.

### 2.5 Depression (PHQ-9)

The 9-item Patient Health Questionnaire (PHQ-9) scale was used to assess depressive symptoms. Each item is scored from 0 (none at all) to 3 (almost every day). The total score ranges from 0 to 27 points, with higher scores indicating more severe depressive symptoms. Scores of 5, 10, 15, and 20 points correspond to mild, moderate, moderate and severe, and major depressive disorder (50, 51). The Chinese version of the PHQ-9 has demonstrated good reliability and validity, with a Cronbach’s α of 0.86 (52). A total score of 10 or higher was considered indicative of significant depressive symptoms (53, 54). The Cronbach’s α in this study was 0.898.

### 2.6 Perceived school atmosphere (PSCI-M)

The Perceived School Climate Inventory-M (PSCI-M) includes five factors: teacher-student relationship, peer relationship, academic pressure, order and discipline, and developmental diversity (55). This study utilized two factors: teacher-student relationship and classmate relationship, with a total of 16 items. The PSCI-M employs a four-point rating from 1 (very inconsistent) to 4 (very consistent). The Cronbach’s α in this study was 0.917. The scoring ranges for teacher-student relationship and peer relationship are 9-36 (scores > 18 were defined as “good”) and 8-32 (scores > 14 were defined as “good”), respectively.

### 2.7 Family functioning (FAD)

The general functioning subscale of the Family Assessment Device (FAD) is a 12-item measure that assesses overall family functioning (56). The total score ranges from 0 to 4 and is categorized into low, medium, and high levels based on the median. Scores greater than or equal to 1 but less than 2 are rated as “low,” scores from 2 to less than 3 are rated as “medium,” and scores of 3 or higher are rated as “high.” The Cronbach’s α in college students was reported as 0.915 (57). In our sample, the Cronbach’s α was 0.697.

### 2.8 Covariates

Participants provided demographic information, including gender, age, grade, whether they were an only child, and the occupation and educational level of their parents. Additional data were collected on smoking history, school residency experience, primary caregivers during their stay, and peer relationships. Family environment, perceived school atmosphere, and smoking have been shown to be related to mental health problems (58, 59). Therefore, We classified and included these variables as covariates to adjust our results.

### 2.9 Statistical analysis

Quantitative variables, given their non-normal distribution, were characterized using the median and interquartile range (IQR). Categorical variables were represented by counts and percentages.

To discern inter-group differences, chi-square tests were applied to categorical variables. For non-normally distributed continuous variables, such as age, Kruskal-Wallis tests were employed.

To assess the association between mental health symptoms and bullying victimization in LBC, multivariate logistic regression analyses were conducted. These analyses were adjusted for various covariates across different models. Odds ratios (OR) were reported along with their corresponding 95% confidence intervals (CI), and statistical significance was considered at p<0.05. All analyses were performed using R software, version 4.2.2.

## 3 Results

### 3.1 Differences between LBC and non-LBC

Among the 28,036 students, 5,114 (18.2%) had left-behind experiences. The demographic characteristics and prior experience of LBC and control groups are detailed in **Table 1**. Significant differences between the two groups were observed in terms of grades, caregivers, and the occupation and educational level of parents, while no significant gender differences were found. The average age of LBC was 14.05 (SD=2.33), compared to 13.47 (SD=2.40) for non-LBC. Most LBC were in high school (41.2%), whereas non-LBC were predominantly in junior high school (36.9%). Throughout their growth, the primary caregivers for LBC were mainly grandparents (49.6%), while non-LBC were parents (42.4%). Among the surveyed students, a majority of LBC’s parents were blue collar workers (Paternal: 32.5%; Maternal: 33.6%), whereas non-LBC’s parents were predominantly farmers (Paternal: 22.1%; Maternal: 24.1%). Additionally, a smaller proportion of LBC’s parents had a college degree or above (Paternal: 5.7%; Maternal: 4.3%) compared to non-LBC’s parents (Paternal: 11.9%; Maternal: 8.7%).

**Table 1:** Differences between LBC and non-LBC, stratified by demographic characteristics and mental health status.

Variable	non-LBC	LBC	p-value
	(n = 22922)	(n = 5114)	
	n (%)	n (%)	
Gender			
Female	11587 (50.5)	2537 (49.6)	0.23
Male	11335 (49.5)	2577 (50.4)	
Age (median[IQR])	14.00[12.00, 16.00]	14.00[12.00, 16.00]	<0.001
Grade			
High school	7718 (33.7)	2107 (41.2)	<0.001
Junior high school	8463 (36.9)	1744 (34.1)	
Primary school	5996 (26.2)	973 (19.0)	
Vocational school	745 (3.3)	290 (5.7)	
Caregivers			
Both Parents	9721 (42.4)	981 (19.2)	<0.001
Father only	8174 (35.7)	1005 (19.7)	
Grandparents	3294 (14.4)	2534 (49.6)	
Mother only	1414 (6.2)	352 (6.9)	
Other close relatives	319 (1.4)	242 (4.7)	
Residence experience			
No	7225 (31.5)	1200 (23.5)	<0.001
Yes	15697 (68.5)	3914 (76.5)	
Only child			
No	14242 (62.1)	3296 (64.5)	0.002
Yes	8680 (37.9)	1818 (35.5)	
Smoking history			
No	22599 (98.6)	4991 (97.6)	<0.001
Yes	323 (1.4)	123 (2.4)	
Paternal education			
College and above	2718 (11.9)	291 (5.7)	<0.001
Primary school	3714 (16.2)	1073 (21.0)	
Secondary school	15798 (68.9)	3570 (69.8)	
Unknown	692 (3.0)	180 (3.5)	
Paternal occupation ^a^			
Blue collar	4823 (21.0)	1664 (32.5)	<0.001
Farmer	5066 (22.1)	1026 (20.1)	
Others	4137 (18.0)	1066 (20.8)	
Self-employed	4692 (20.5)	672 (13.1)	
White collar	4204 (18.3)	686 (13.4)	
Maternal education			
College and above	1990 (8.7)	218 (4.3)	<0.001
Primary school	4986 (21.8)	1371 (26.8)	
Secondary school	15087 (65.8)	3182 (62.2)	
Unknown	859 (3.7)	343 (6.7)	
Maternal occupation ^a^			
Blue collar	5120 (22.3)	1716 (33.6)	<0.001
Farmer	5533 (24.1)	1021 (20.0)	
Others	5171 (22.6)	1274 (24.9)	
Self-employed	4585 (20.0)	672 (13.1)	
White collar	2513 (11.0)	431 (8.4)	
Verbal bullying			
Often	2237 (9.8)	667 (13.0)	<0.001
Seldom	20685 (90.2)	4447 (87.0)	
Physical bullying			
Often	1144 (5.0)	377 (7.4)	<0.001
Seldom	21778 (95.0)	4737 (92.6)	
Relational bullying			
Often	1556 (6.8)	486 (9.5)	<0.001
Seldom	21366 (93.2)	4628 (90.5)	
Cyber bullying			
Often	565 (2.5)	194 (3.8)	<0.001
Seldom	22357 (97.5)	4920 (96.2)	
Any bullying			
Often	2773 (12.1)	832 (16.3)	<0.001
Seldom	20149 (87.9)	4282 (83.7)	
Teacher-student relationship			
Good	21651 (94.5)	4744 (92.8)	<0.001
Poor	1271 (5.5)	370 (7.2)	
Peer relations			
Good	21827 (95.2)	4784 (93.5)	<0.001
Poor	1095 (4.8)	330 (6.5)	
Family function			
High	13235 (57.7)	2317 (45.3)	<0.001
Low	395 (1.7)	160 (3.1)	
Medium	9292 (40.5)	2637 (51.6)	
Self-injurious behavior			
For suicide	656 (2.9)	253 (4.9)	<0.001
No	20140 (87.9)	4207 (82.3)	
Not for suicide	2126 (9.3)	654 (12.8)	
Depressive symptoms			
No	18486 (80.6)	3686 (72.1)	<0.001
Yes	4436 (19.4)	1428 (27.9)	
Anxiety symptoms			
No	18795 (82.0)	3889 (76.0)	<0.001
Yes	4127 (18.0)	1225 (24.0)	

^a^Paternal and maternal occupation were defined as blue collar (including professional skill worker, commercial and service worker, industrial worker), white collar (including cadres of agencies, enterprises and institutions, teacher, soldier), self-employed (defined as individual proprietors and private entrepreneurs), farmer and others (including retired, unemployed, semi-unemployed individuals and others).

Compared with non-LBC, LBC reported worse interpersonal relationships, both with peers (non-LBC: 4.8%; LBC: 6.5%) and teachers (non-LBC: 5.5%; LBC: 7.2%). Additionally, a higher proportion of LBC had residence experience (76.5%) and a smoking history (2.4%). LBC also exhibited a higher frequency of bullying problems (LBC: 16.3%; non-LBC: 12.1%).

We also compared the prevalence of anxiety, depressive symptoms and self-injurious behavior between LBC and non-LBC. LBC exhibited a higher prevalence of anxiety symptoms (24.0% vs. 18.0%), depressive symptoms (27.9% vs. 19.4%), and self-injurious behavior (17.7% vs. 12.2%). Meanwhile, the proportion of LBC with suicidal purposes (4.9% vs. 2.9%) and non-suicidal purposes (12.8% vs. 9.3%) was higher compared to non-LBC.

### 3.2 The mental health status of LBC

**Table 2** compared the prevalence of mental health problem among LBC by stratifying them according to demography information, caregivers, and different types of bullying (including verbal, physical, relational, and cyber bullying). Among LBC, girls were more likely to experience anxiety, depressive symptoms, and self-injurious behavior.

**Table 2:** Mental health among LBC (n=5114) after stratification by demographic characteristics and life experience.

Variable	Anxiety symptoms		p-value	Depressive symptoms		p-value	Self-injurious behavior		p-value
	n(yes)	%		n(yes)	%		n(yes)	%	
	1225	24.0		1428	27.9		907	17.7	
Gender									
Male	553	21.5	<0.001	640	24.8	<0.001	375	14.6	<0.001
Female	672	26.5		788	31.1		532	21.0	
Grade									
High school	545	25.9	<0.001	711	33.7	<0.001	464	22.0	<0.001
Junior high school	453	26.0		479	27.5		294	16.9	
Primary school	146	15.0		135	13.9		70	7.2	
Vocational school	81	27.9		103	35.5		79	27.2	
LBC Caregivers									
Brother or sister	35	31.8	0.046	37	33.6	0.011	23	20.9	<0.001
Father	63	26.7		59	25.0		63	26.7	
Grandparents	816	23.5		941	27.1		568	16.4	
Mother	207	24.4		247	29.1		149	17.5	
Other close relatives	77	20.9		109	29.5		84	22.8	
Others	27	33.8		35	43.8		20	25.0	
Smoking history									
No	1185	23.7	0.032	1377	27.6	0.001	843	16.9	<0.001
Yes	40	32.5		51	41.5		64	52.0	
Verbal bullying									
Seldom	882	19.8	<0.001	1024	23.0	<0.001	661	14.9	<0.001
Often	343	51.4		404	60.6		246	36.9	
Physical bullying									
Seldom	1021	21.6	<0.001	1192	25.2	<0.001	759	16.0	<0.001
Often	204	54.1		236	62.6		148	39.3	
Relational bullying									
Seldom	970	21.0	<0.001	1127	24.4	<0.001	717	15.5	<0.001
Often	255	52.5		301	61.9		190	39.1	
Cyber bullying									
Seldom	1119	22.7	<0.001	1288	26.2	<0.001	812	16.5	<0.001
Often	106	54.6		140	72.2		95	49.0	
Any bullying									
Seldom	818	19.1	<0.001	948	22.1	<0.001	613	14.3	<0.001
Often	407	48.9		480	57.7		294	35.3	
Teacher-student relationship									
Good	1091	23.0	<0.001	1265	26.7	<0.001	786	16.6	<0.001
Poor	134	36.2		163	44.1		121	32.7	
Peer relations									
Good	1100	23.0	<0.001	1272	26.6	<0.001	805	16.8	<0.001
Poor	125	37.9		156	47.3		102	30.9	
Family function									
High	348	15.0	<0.001	362	15.6	<0.001	229	9.9	<0.001
Low	82	51.3		112	70.0		77	48.1	
Medium	795	30.1		954	36.2		601	22.8	

Among LBC, the prevalence of depressive symptoms and self-injurious behavior is higher among those in high school (depressive symptoms: 33.7%; self-injurious: 22.0%) and vocational school (depressive symptoms: 35.5%; self-injurious: 27.2%). LBC whose primary caregiver is their father are more likely to engage in self-injurious behavior (26.7%). Anxiety (33.8%, p<0.05) and depressive symptoms (43.8%, p<0.05) are more prevalent among those with primary caregivers identified as “others”, with brothers or sisters following (anxiety symptoms: 31.8%, p<0.05; depressive symptoms: 33.6%, p<0.05).

LBC with a history of smoking exhibit higher rates of anxiety (32.5% vs. 23.7%), depressive symptoms (41.5% vs. 27.6%) and self-injurious behavior (52.0% vs. 16.9%). LBC who are often bullied show higher rates of anxiety (48.9% vs. 19.1%) and depressive symptoms (57.7% vs. 22.1%) compared to those who are seldom or never bullied, and are more likely to engage in self-injurious behavior (35.3% vs. 14.3%). Compared to other types of bullying, LBC who frequently experience cyberbullying have higher prevalence rates of depressive symptoms (72.2%) and self-injurious behavior (49.0%). Additionally, LBC with poor teacher-student relationships (anxiety symptoms: 36.2%; depressive symptoms: 44.1%; self-injurious: 32.7%) and poor peer relationships (anxiety symptoms: 37.9%; depressive symptoms: 47.3%; self-injurious: 30.9%) also exhibit a higher incidence of mental health problems.

### 3.3 Mental health risks of different types of bullying

**Table 3** examined the effects of different types of bullying on anxiety, depressive symptoms, and self-injurious behavior among LBC. Among LBC, physical bullying showed the most significant positive correlation with anxiety symptoms (OR 2.03, 95% CI 1.54-2.67; Anxiety symptoms - Model B, **Table 3**). Even after adjusting for the effects of different types of bullying, physical bullying remains a significant predictor of anxiety symptoms (OR 1.62, 95% CI 1.11-2.38; Anxiety symptoms - Model C, **Table 3**).

**Table 3:** The contribution of different types of bullying to anxiety symptoms, depression symptoms and self-injurious behavior among LBC.

Variable	Anxiety symptoms			Depressive symptoms			Self-injurious behavior		
	Model A^a^	Model B^b^	Model C^c^	Model A^a^	Model B^b^	Model C^c^	Model A^a^	Model B^b^	Model C^c^
	OR (95%CI)	OR (95%CI)	OR (95%CI)	OR (95%CI)	OR (95%CI)	OR (95%CI)	OR (95%CI)	OR (95%CI)	OR (95%CI)
Any bullying									
Often	4.52(3.85-5.32)	1.89(1.55-2.32)	NA	5.92(5.01-7.00)	3.29(2.68-4.04)	NA	3.85(3.23-4.60)	2.17(1.78-2.63)	NA
Seldom	Ref.	Ref.		Ref.	Ref.		Ref.	Ref.	
Verbal bullying									
Often	4.66(3.91-5.55)	1.91(1.54-2.38)	1.54(1.15-2.06)	6.07(5.07-7.29)	3.29(2.64-4.11)	2.23(1.66-3.00)	3.81(3.16-4.60)	2.05(1.66-2.53)	1.54(1.16-2.04)
Seldom	Ref.	Ref.	Ref.	Ref.	Ref.	Ref.	Ref.	Ref.	Ref.
Physical bullying									
Often	5.13(4.11-6.43)	2.03(1.54-2.67)	1.62(1.11-2.38)	6.51(5.16-8.24)	3.24(2.44-4.33)	1.08(0.72-1.61)	4.29(3.38-5.43)	2.09(1.61-2.72)	1.12(0.77-1.62)
Seldom	Ref.	Ref.	Ref.	Ref.	Ref.	Ref.	Ref.	Ref.	Ref.
Relational bullying									
Often	4.66(3.82-5.68)	1.86(1.45-2.38)	1.37(0.97-1.92)	6.14(5.00-7.57)	3.29(2.55-4.26)	1.52(1.07-2.15)	4.11(3.32-5.07)	2.12(1.68-2.68)	1.33(0.95-1.85)
Seldom	Ref.	Ref.	Ref.	Ref.	Ref.	Ref.	Ref.	Ref.	Ref.
Cyber bullying									
Often	4.44(3.30-5.98)	1.35(0.94-1.94)	0.54(0.33-0.86)	8.19(5.92-11.49)	4.94(3.31-7.42)	1.86(1.12-3.12)	5.38(3.96-7.32)	2.48(1.77-3.46)	1.36(0.88-2.11)
Seldom	Ref.	Ref.	Ref.	Ref.	Ref.	Ref.	Ref.	Ref.	Ref.

CI, confidence interval; NA, not applicable; OR, odds ratio; Ref., reference.

a. ORs were adjusted for sex, age, grade, paternal occupation, maternal occupation, paternal education, maternal education. Due to the previous analysis finding that there was no significant difference in the prevalence of anxiety among parents with different educational backgrounds, paternal/maternal education level was not included as a covariate in the anxiety model.

b. ORs were additionally adjusted for caregivers, residence experience, smoking history, teacher student relationship, peer relations, and some other mental health problems.

c. ORs were additionally adjusted for other 3 types of bullying.

There was a significant positive correlation between various types of bullying and depressive symptoms among LBC. After adjusting for the effects of different types of bullying, verbal bullying emerged as the most significant predictor of depressive symptoms (OR 2.23, 95% CI 1.66-3.00; Depressive symptoms - Model C, **Table 3**). This was followed by cyber bullying (OR 1.86, 95% CI 1.12-3.12; Depressive symptoms - Model C, **Table 3**) and relational bullying (OR 1.52, 95% CI 1.07-2.15; Depressive symptoms - Model C, **Table 3**).

We also observed a positive correlation between bullying and self-injurious behavior among LBC, with the most significant association found for cyber bullying (OR 2.48, 95% CI 1.77-3.46; Self-injurious behavior - Model B, **Table 3**). After adjusting for different types of bullying, significant associations were observed only for verbal bullying (OR 1.54, 95% CI 1.16-2.04; Self-injurious behavior - Model C, **Table 3**).

### 3.4 The impact of bullying victims

**Table 4** compared the differences between LBC and non-LBC among those who have experienced bullying. The proportion of self-injurious behavior was significantly higher among LBC (for suicide: 14.3%; not for suicide: 21.0%) compared to non-LBC (for suicide: 9.4%; not for suicide: 18.6%), regardless of the intent. Additionally, the prevalence of anxiety (48.9% vs. 44.5%) and depressive symptoms (57.7% vs. 49.6%) was higher among LBC. The severity scores for both anxiety and depressive symptoms were also significantly elevated in LBC compared to non-LBC. Furthermore, the overall family function of non-LBC is higher than that of LBC.

**Table 4:** Compared the differences between left-behind and non-left-behind students who have been bullied.

Variable	non-LBC	LBC	p-value
	(n = 2773)	(n = 832)	
	n (%)	n (%)	
Teacher-student relationship			
Good	2458 (88.6)	725 (87.1)	0.263
Poor	315 (11.4)	107 (12.9)	
Peer relations			
Good	2381 (85.9)	702 (84.4)	0.311
Poor	392 (14.1)	130 (15.6)	
Family function			
High	965 (34.8)	240 (28.8)	0.001
Low	149 (5.4)	65 (7.8)	
Medium	1659 (59.8)	527 (63.3)	
Self-injurious behavior			
For suicide	260 (9.4)	119 (14.3)	<0.001
No	1996 (72.0)	538 (64.7)	
Not for suicide	517 (18.6)	175 (21.0)	
Depression level			
No depression	653 (23.5)	148 (17.8)	0.001
Mild depression	745 (26.9)	204 (24.5)	
Moderate depression	645 (23.3)	225 (27.0)	
Moderate and severe depression	459 (16.6)	163 (19.6)	
Severe depression	271 (9.8)	92 (11.1)	
Anxiety level			
No anxiety	618 (22.3)	147 (17.7)	0.024
Presence of symptoms	920 (33.2)	278 (33.4)	
Moderate anxiety	767 (27.7)	259 (31.1)	
Severe anxiety	468 (16.9)	148 (17.8)	

### 3.5 Protective factors for the mental health of LBC

**Table 5** categorized LBC into four groups based on their experiences with bullying and mental health problems. For LBC who have experienced both bullying and mental health problems (Group 1), good peer relationships (OR 0.35, 95% CI 0.26-0.49, p<0.001) and higher family function (High: OR 0.14, 95% CI 0.09-0.20, p<0.001; Medium: OR 0.36, 95% CI 0.25-0.52, p<0.001) serve as protective factors. Among LBC who did not experience bullying but had mental health problems (Group 2), a good teacher-student relationship (OR 0.76, 95% CI 0.58-0.99, p=0.047) was identified as a protective factor. LBC who had neither experienced bullying nor mental health problems (Group 4) demonstrated that good peer relationships (OR 2.06, 95% CI 1.54-2.79, p<0.001) and higher family function (High: OR 11.47, 95% CI 7.34-18.76, p<0.001; Medium: OR 4.43, 95% CI 2.84-7.22, p<0.001) were associated with better outcomes. Details are shown in **Table 5**.

**Table 5:** Multinomial logistic regression to independent risk factors for bullying and mental health symptoms among LBC.

Variable	Group1 ^a^	p-value	Group2 ^b^	p-value	Group3 ^c^	p-value	Group4 ^d^	p-value
	OR (95%CI)		OR (95%CI)		OR (95%CI)		OR (95%CI)	
Age	0.97 (0.88, 1.07)	0.59	1.17 (1.09, 1.25)	<0.001	0.84 (0.72, 0.97)	<0.05	0.92 (0.86, 0.98)	<0.05
Gender								
Female	1.00		1.00		1.00		1.00	
Male	1.18 (0.99, 1.41)	0.07	0.60 (0.53, 0.68)	<0.001	1.54 (1.16, 2.05)	<0.05	1.33 (1.18, 1.49)	<0.001
Grade								
Primary school	1.00		1.00		1.00		1.00	
High school	1.00 (0.55, 1.83)	0.99	1.46 (0.93, 2.29)	0.10	0.85 (0.34, 2.12)	0.72	0.86 (0.58, 1.29)	0.48
Junior high school	1.47 (1.01, 2.16)	<0.05	1.44 (1.06, 1.95)	<0.05	0.71 (0.41, 1.23)	0.22	0.80 (0.62, 1.03)	0.08
Vocational school	1.26 (0.64, 2.46)	0.50	1.58 (0.96, 2.61)	0.07	1.30 (0.46, 3.64)	0.62	0.69 (0.44, 1.09)	0.11
Paternal education								
Primary school	1.00		1.00		1.00		1.00	
College and above	1.26 (0.82, 1.91)	0.29	0.86 (0.61, 1.22)	0.42	1.38 (0.71, 2.60)	0.33	0.96 (0.70, 1.31)	0.78
Secondary school	0.81 (0.65, 1.01)	0.06	1.05 (0.89, 1.24)	0.55	0.95 (0.66, 1.38)	0.77	1.08 (0.92, 1.26)	0.34
Unknown	1.56 (0.97, 2.45)	0.06	0.73 (0.48, 1.08)	0.12	1.30 (0.56, 2.76)	0.51	0.96 (0.67, 1.37)	0.82
Maternal education								
Primary school	1.00		1.00		1.00		1.00	
College and above	0.86 (0.53, 1.38)	0.54	0.93 (0.63, 1.36)	0.72	1.11 (0.54, 2.18)	0.77	1.06 (0.75, 1.49)	0.76
Secondary school	0.84 (0.68, 1.04)	0.12	0.93 (0.80, 1.09)	0.38	0.79 (0.56, 1.11)	0.17	1.19 (1.03, 1.37)	<0.05
Unknown	0.71 (0.47, 1.06)	0.10	0.96 (0.71, 1.28)	0.78	0.73 (0.37, 1.37)	0.35	1.31 (0.99, 1.72)	0.06
Paternal occupation								
Blue collar	1.00		1.00		1.00		1.00	
Farmer	1.09 (0.77, 1.54)	0.63	0.66 (0.51, 0.86)	<0.05	1.59 (0.95, 2.66)	0.08	1.24 (0.98, 1.56)	0.07
Others	1.05 (0.78, 1.40)	0.75	1.06 (0.86, 1.31)	0.59	0.72 (0.43, 1.18)	0.20	0.98 (0.81, 1.20)	0.88
Self-employed	0.99 (0.68, 1.43)	0.95	0.93 (0.71, 1.21)	0.60	1.17 (0.66, 2.03)	0.58	1.04 (0.81, 1.33)	0.75
White collar	1.10 (0.81, 1.51)	0.53	1.00 (0.79, 1.27)	0.99	0.95 (0.57, 1.55)	0.84	0.97 (0.78, 1.21)	0.79
Maternal occupation								
Blue collar	1.00		1.00		1.00		1.00	
Farmer	0.76 (0.53, 1.09)	0.14	1.27 (0.99, 1.64)	0.06	0.94 (0.55, 1.61)	0.81	0.92 (0.73, 1.17)	0.51
Others	0.91 (0.69, 1.21)	0.52	1.16 (0.94, 1.42)	0.16	1.25 (0.79, 1.97)	0.34	0.89 (0.73, 1.07)	0.22
Self-employed	0.70 (0.47, 1.03)	0.07	1.08 (0.82, 1.41)	0.58	1.05 (0.59, 1.83)	0.87	1.08 (0.85, 1.39)	0.52
White collar	1.15 (0.81, 1.64)	0.42	1.07 (0.80, 1.42)	0.64	1.12 (0.62, 1.99)	0.69	0.85 (0.65, 1.09)	0.20
LBC Caregivers								
Mother	1.00		1.00		1.00		1.00	
Brother or sister	1.42 (0.79, 2.43)	0.22	0.93 (0.59, 1.46)	0.77	0.21 (0.01, 0.99)	0.13	0.99 (0.65, 1.53)	0.97
Father	1.04 (0.68, 1.59)	0.84	0.96 (0.68, 1.33)	0.80	0.52 (0.19, 1.18)	0.15	1.12 (0.82, 1.53)	0.49
Grandparents	0.84 (0.66, 1.08)	0.17	0.87 (0.73, 1.04)	0.13	1.14 (0.79, 1.68)	0.50	1.18 (1.00, 1.39)	0.05
Other close relatives	0.89 (0.60, 1.30)	0.55	1.02 (0.77, 1.35)	0.87	0.81 (0.40, 1.53)	0.53	1.06 (0.81, 1.38)	0.67
Others	1.26 (0.63, 2.35)	0.49	1.02 (0.61, 1.69)	0.94	0.91 (0.21, 2.67)	0.88	0.87 (0.52, 1.43)	0.58
Residence experience								
Yes	1.00		1.00		1.00		1.00	
No	1.28 (1.01, 1.62)	<0.05	0.95 (0.79, 1.15)	0.62	0.92 (0.64, 1.31)	0.66	0.92 (0.78, 1.09)	0.35
Smoking history								
Yes	1.00		1.00		1.00		1.00	
No	0.69 (0.44, 1.11)	0.11	0.55 (0.37, 0.81)	<0.05	1.45 (0.58, 4.87)	0.48	2.47 (1.61, 3.87)	<0.001
Teacher-student relationship								
Poor	1.00		1.00		1.00		1.00	
Good	0.96 (0.69, 1.35)	0.81	0.76 (0.58, 1.00)	<0.05	1.16 (0.63, 2.24)	0.64	1.3 (0.99, 1.72)	0.06
Peer relations								
Poor	1.00		1.00		1.00		1.00	
Good	0.35 (0.26, 0.49)	<0.001	1.27 (0.95, 1.73)	0.11	0.54 (0.31, 0.97)	<0.05	2.06 (1.54, 2.79)	<0.001
Family function								
Low	1.00		1.00		1.00		1.00	
High	0.14 (0.09, 0.20)	<0.001	0.34 (0.24, 0.48)	<0.001	0.99 (0.45, 2.62)	0.98	11.47 (7.34, 18.76)	<0.001
Medium	0.36 (0.25, 0.52)	<0.001	0.7 (0.50, 0.98)	<0.05	1.16 (0.53, 3.04)	0.74	4.43 (2.84, 7.22)	<0.001

Age, gender, grade, paternal education, maternal education, paternal occupation, maternal occupation, caregivers during left-behind period, residence experience and smoking history were included as covariates in the model.

^a^Group1 was LBC who experienced any one or several mental health problems such as anxiety, depression, or self-injurious behavior after experiencing bullying victimization.

^b^Group2 was LBC who did not experience bullying victimization but experienced any one or several mental health problems such as anxiety, depression, or self-injurious behavior.

^c^Group3 was LBC who did not experience anxiety, depression, or self-injurious behavior after experiencing bullying victimization.

^d^Group4 was LBC who did not experience bullying victimization and did not experience anxiety, depression, or self-injurious behavior after experiencing bullying victimization.

## 4 Discussion

This study systematically examined the prevalence of mental health issues among left-behind children (LBC) and non-LBC in China, focusing on the impact of bullying victimization on anxiety, depression, and self-injurious behaviors. Our findings showed that LBC experienced higher rates of bullying and were more prone to anxiety, depression, and self-injury compared to non-LBC. Among bullied LBC, nearly half displayed anxiety and depressive symptoms, and over one-third engaged in self-harm. The study identified physical bullying as a major predictor of anxiety, while verbal bullying was significantly linked to depression and self-injury. Additionally, LBC showed higher symptom severity and self-injurious behaviors, especially with suicidal intent. Notably, higher family functioning and positive teacher-student and peer relationships offered protective effects against mental health problems in bullied LBC. These findings underscore the need for early interventions to support the mental health of children and adolescents, especially those who are left behind.

Our research indicates that LBC is more susceptible to psychological health problems, including anxiety, depressive symptoms, and self-injurious behavior, aligning with previous findings on the heightened vulnerability of LBC to mental illness (17, 21). The absence of parental protection increases the susceptibility of LBC to feelings of despair and loneliness, which can gradually evolve into feelings of inferiority (60). Existing studies have also reported a higher risk of low self-esteem in LBC, which is one of the most powerful clinical predictors of depression (61). LBC shows more emotional problems, such as loneliness and inferiority, as well as a higher incidence of emotional disorders. The suppression of their emotions may exacerbate mental health problems and potentially lead to suicidal behavior (62). During this process, sufficient care and support from parents can largely eliminate these negative emotions. However, a sustained lack of such support can lead to an escalation of negative emotions, potentially resulting in internalizing problematic behaviors (2).

Our results found that the victimization rate of bullying among children and adolescents is 12.9%. This finding aligns with the range of reported bullying victimization rates in China in recent years (28, 29). Consistent with previous research, our study found that LBC who experienced bullying had a higher incidence of anxiety and depressive symptoms (35). Each type of bullying victimization was associated with anxiety and depressive symptoms among adolescents, emphasizing the detrimental effects of bullying in the development of mental health in adolescents. A longitudinal study conducted in Norway similarly found that experiences of bullying in adolescents could predict subsequent anxiety and depressive symptoms (63). The relationships between various types of bullying victimizations and mental health problems will be discussed below.

The incidence of bullying among LBC was significantly higher than in non-LBC, particularly with respect to verbal bullying. The victimization of LBC bullying is largely related to their living environment. Prolonged lack of parental care and supervision increases their susceptibility to bullying, which may lead to more frequent incidents of bullying (64). Meanwhile, LBC typically experience stronger feelings of loneliness, which also reduces their likelihood of receiving help from others when being bullied (65). There is a strong correlation between bullying and self-esteem levels. Reduced parental support may contribute to a higher likelihood of low self-esteem (21). Existing studies have shown that self-esteem serves as a partial mediator between social anxiety and school bullying victimization, as well as between school life satisfaction and school bullying victimization (66). Improving the level of self-esteem may help reduce LBC’s chances of being bullied (67).

A significant impact of bullying on depressive symptoms was found in our study. Verbal bullying is often accompanied by insults and disapproval from others. According to Beck’s diathesis-stress theory (68), bullying victimization would be considered a stressful life event that can activate cognitive vulnerabilities (specifically self-esteem), leading to significant negative outcomes. Verbal bullying, compared to other types of bullying, has been found to have a greater impact on depressive symptoms and self-injurious behavior. The hopelessness theory of depression, a cognitive diathesis-stress model of depression, proposed that negative life events could lead to negative self-inferences, which in turn, may serve as a proximal contributor to hopelessness-based depression (69). Changes in children’s negative self-perceptions can be influenced by negative evaluations from others. Alterations in self-esteem levels, resulting from these negative self-evaluations, mediate the relationship between negative evaluations in verbal bullying and the development of depressive symptoms (70, 71).

In our study, physical bullying emerged as the primary risk factor for anxiety symptoms. Physical bullying involves threats or injuries to an individual’s body and damage to personal property.

Research on interpersonal violence has identified somatic symptoms, such as headaches, chronic abdominal pain, and sleep disorders, as common consequences of physical and sexual abuse, bullying, and adolescent violence in children (72). A meta-analysis indicated that individuals targeted by bullying are at a higher risk for anxiety, depression, alcohol misuse, and substance abuse compared to those who are not targeted (37). Physical bullying poses a direct threat to the physical safety of children. Pain memory resulting from bullying is a multidimensional subjective experience, encompassing sensation (e.g., pain intensity), emotion (e.g., fear-related emotions), and contextual factors (e.g., time, place, and individuals involved) (73, 74). The encoding of pain stimuli involves the medial prefrontal cortex (mPFC) and the anterior cingulate gyrus (ACC) (75). Under the influence of situational factors, there is an overlap between pain and fear systems, resulting in avoidance behaviors (76). This overlap increases the likelihood of anxiety disorders, which are characterized by symptoms of anxiety, fear, nervousness and worry (77).

The association between family functioning and mental health is consistent with previous research, indicating that students with high family functioning are at a lower risk for developing mental health problems, including anxiety and depression symptoms (62, 78). Olson’s circular pattern theory posits that family functioning encompasses emotional connections, family rules, communication, and the effectiveness of coping with external events within the family system (79). The theory identifies three dimensions of family functioning: family intimacy, family adaptability, and family communication. Studies have shown that a strong parent-child relationship within the family environment effectively reduces the risk of emotional and behavioral problems, thereby mitigating the negative impact of parental absence on LBC (80). Conversely, adolescents who experience communication difficulties with their parents are more susceptible to psychological problems (81). Empirical analysis of longitudinal data revealed the crucial role of parent-child relationships in individual personality development, with positive relationships and support from parents helping to prevent potential negative emotional effects (11). This implies the need to foster emotional and informational understanding among family members through proactive communication practices, such as active listening, empathy, and supportive language, along with engaging in family activities. Such practices strengthen the resilience of families and individuals in the face of environmental changes and partially offset any functional deficiencies within the family (42, 80).

Consistent with previous studies, positive teacher-student and peer relationships within the school environment have been found to benefit the mental health of LBC who experience bullying (82, 83). Enhanced interpersonal quality reduces the persistence of bullying by encouraging bullied students to actively seek help (84). Additionally, supportive teacher-student relationships and peer relationships can reduce loneliness experiences by improve self-identity (85). Previous studies have demonstrated the importance of strong social connections and resources for mental health following adverse events, as they can provide motivation, alleviate loneliness, and bolster resilience during stressful situations or life adversities (86, 87). The development of supportive interpersonal relationships and accurate self-awareness in children requires significant attention and emphasis from both society and educational institutions.

In summary, our research provides valuable insights for the screening and intervention of mental health problems among children and adolescents. In addition to the above suggestions, schools and relevant education departments should promote routine mental health assessments and enhance both the evidence base and data accessibility. Advance the application of artificial intelligence in screening, monitoring and service provision within the realm of mental health.

## 5 Strengths and Limitations

Our study examined developmental changes in children and adolescents aged 8-19 years to provide guidance for early prevention and intervention throughout their growth. Meanwhile, according to the ecological systems theory (88), we considered the impact of family, school and significant others on the mental health of children and adolescents and included these variables in our analysis. Our results also reveal that self-injurious behaviors with suicidal intent are more prevalent among LBC. his underscores the need for policymakers and mental health professionals to implement comprehensive mental health screening and care services for LBC and other children in similar environments. Several limitations should be noted. First, our study is based on a cross-sectional survey. Longitudinal data is still needed to establish causal relationships between bullying victimization and mental health problems. Second, our study focused solely on bullying victimization without addressing other forms of bullying. In addition, while previous research highlights the significance of family environment and economic status on LBC’s physical and mental health (62), we did not collect data on confounding factors such as family economic status, parenting styles, and self-evaluation. Future studies should aim to include these factors for a more comprehensive analysis.

## 6 Conclusions

Our research indicates that the incidence of mental health problems among Chinese LBC is relatively high. Specifically, LBC with a history of smoking and those whose primary caregivers are not parents, grandparents, or siblings warrant special attention. Furthermore, as a significant risk factor for mental health problems, bullying victimization necessitates attention due to its detrimental effects: physical bullying exacerbates anxiety symptoms, verbal bullying contributes to depressive symptoms, and self-injurious behaviors. High family functioning and positive school interpersonal relationships are crucial protective factors in the psychological development of children and adolescents. Future studies should develop various risk prediction models for a broader range of mental health problems, focusing on psychological interventions tailored to the specific characteristics of different populations.

## Data Availability

The raw data supporting the conclusions of this article will be made available by the authors, without undue reservation.
